# The effects of ex vivo cold-storage on cryopreservation of the goat (Caprus hircus) epididymal sperm

**Published:** 2013-09

**Authors:** Seyed Kamal-Aldin Hoseinzadeh-Sani, Farid Barati, Mahmoud Khaksary Mahabady

**Affiliations:** 1 *Department of Clinical Sciences, Faculty of Veterinary Medicine, Shahid Chamran University of Ahvaz, Ahvaz, Iran. *; 2 *Department of Basic Sciences, Faculty of Veterinary Medicine, Shahid Chamran University of Ahvaz, Ahvaz, Iran.*

**Keywords:** Epididymal sperm retrieval, Cryopreservation, Refrigeration, Cold-storage, Goat

## Abstract

**Background:** There are many studies focused on long or short storage time of epididymal sperm (EPS) of different species. There are limited studies on preservation or cryopreservation of the domestic goat EPS.

**Objective: **The aim of the present study was to evaluate the effects of ex vivo cold-storage on freezing of EPS from goat (Capra hircus).

**Materials and Methods:** In a split-plot design the caprine testes-epididymides (40 pairs) were divided to 4 storage-time groups equally (0, 24, 48 and 72 h), then subjected to cryopreservation using Bioxell. Sperm parameters were analyzed before and after freezing.

**Results: **Duration of cold-storage as well as freezing at all storage-time points reduced sperm viability and progressive motility while increased sperm tail abnormalities (p<0.0001). Freezing reduced the percentage of cytoplasmic droplets (p<0.0001). The percentage of detached heads was increased at all storage-time points following freezing (p=0.0019), except at time 0 h.

**Conclusion:** It can be concluded that cold storage [in refrigerator (4^o^C) for 72 h] of epididymides efficiently protected the goat EPS in terms of progressive motility and viability. However, cold-storage may not protect the goat EPS against cryopreservation with Bioxell.

## Introduction

Epididymal sperm (EPS), although doesn't have a notable role in artificial insemination, is important in understanding the bases of sperm preservation as a tool for conservation of genetic resources of endangered dead wild life species or assisted technology for treatment of the some cases (e.g. obstructive azoospermia) of human infertilities (1, 2). However, the low quarter neurologic disorders and some extracts may influence the quality of EPS (3, 4). On the other hand, short-time storage of sperm usually is possible above zero temperatures, for manipulation purposes, and may protect the fertility potential of sperm. Short-time storage of the EPS usually, is done within or out of the epididymis in a cool condition (5-7). Pre-freezing poor sperm quality has detrimental effects on the sperm cryopreservation (8). 

The postmortem changes within epididymis can reduce the quality of cool-stored EPS, and in turn decrease the freezing output of the EPS (7, 9). Ganan *et al* showed the efficacy of 24 h cold-storage on the quality and cryopreservation of EPS from domestic cat (6). The fresh EPS of different species has been collected and frozen successfully (7, 10, 11). Sperm handling procedure is critical for optimal sperm freezing output. The basic media as well as the commercial extenders were used for EPS freezing (7, 12-14). The addition of the protective agents and seminal plasma and determination of optimal equilibration time have been considered in EPS cryopreservation, too (15-17). 

The aim of the present study was to find the effects of *ex vivo* cold-storage on cryopreservation of the goat EPS with a known commercial sperm freezing extender.

## Materials and methods

**Reagents and diluents**

Tyrode solution ingredients and sodium lactate were from Merck, Germany. Bovine albumin, sodium pyrovate and eosin-B and nigrosin were bought from Sigma-Aldrich, USA. Bioxell® was provided from IMV Technology, France. Giemsa stain was provided from Baharafshan, Iran.

**Experimental procedures**

*Preparation of Epididymal sperm*

The testes-epididymides were provided from slaughtered bucks (9-13 months of age) at the local slaughterhouse during January to March 2012 (breeding season) and transported on ice to the laboratory of IVF, Shahid Chamran University of Ahvaz, in 1 h. Both left and right cauda-epididymides were separated and a deep incision was placed on their ventral part, and then left in the warmed TALP (Tyrode- Albumin-Lactate-Pyrovate) solution (2 mL) for 15 min to retrieve sperm.

*Sperm analysis*

Equal volumes (≈50 µL) of the stain (Eosin B- nigrosin) with the sperm suspension (fresh or frozen samples) were mixed on a glass slide, smeared and finally dried with a hair dryer. Pink or red sperm considered as dead and white sperm considered as live sperm. The percentages of live sperm were estimated from five separated fields with magnification of X40. Another drop of the sperm suspension was smeared on a glass slide, left to be air-dried, fixed with absolute methanol (Baharafshan, Iran) and finally stained with the Giemsa stain (5%) to see the sperm morphological abnormalities (magnification of ×100).

The sperm head abnormalities were small, tapered, pyriform, round and amorphous. The tail abnormalities were bent tail and coiled tail. Cytoplasmic droplets and the most important midpeice abnormality, detached heads, were considered separately. A drop of sperm suspension was placed on a warmed slide and sperm progressive motility was estimated by observation of four different microscopic fields. The sperm concentration of the suspension was estimated using a modified Neubar chamber; based on the procedure for the red blood cell count. 

*Sperm cryopreservation*

Sperm cryopreservation was carried out according to the instruction manual of IMV technology for freezing of bull semen with Bioxell®. Briefly, the working solution was prepared by mixing 4 to 1 (v/v) of Bioxell® to double distilled water. The sperm suspension was extended in a single step with the working solution. The final concentration of 20×10^6^ sperm/mL of extended sperm was adjusted for freezing. 

The extended sperms were packaged at 4^o^C (0.25 mL straws; IMV technology, France) and equilibrated for 4h at 4^o^C. The equilibrated straws were placed on the liquid nitrogen (LN) vapour (4 cm above LN) for 10 min, then seeding was done by touching one end of straws with a super-cooled forceps (in LN). Finally, straws were plunged in LN and remained, at least, for 48 h and thawed in a 37^o^C (for 30 sec) water bath for analysis.

**Experimental design**

The testes-epididymides (n=40 pairs) were assigned to four storage-time groups (0, 24, 48 and 72h; Ten pairs of testes-epididymides per each time) in a 4^o^C refrigerator. After storage, sperm was collected and frozen. Sperm parameters were analyzed before freezing and after thawing. Two straws per storage time were used for frozen sperm analysis.

**Statistical analysis**

All of the collected data on the sperm parameters in the study were considered in a split plot design which main plot was cold-storage time (0, 24, 48 and 72 h) and subplot was type of analyzed sperms (before freezing or after freezing). The General Linear Model (GLM) procedure in SAS was used to analyze the effects of cold-storage and freezing on the goat EPS (18). The difference between the least square means (LS means) was calculated using *pdiff* statement in SAS. Data expressed as LS means and standard error of mean (SEM).

## Results

Freezing, duration of cold-storage (0h, 24h, 48h and 72h) and their two-way interaction affected (p<0.0001) the percentage of viable spermatozoa. Cold-storage also can protect the goat epididymal sperm up to 24h, however the values of 48 and 72 h are acceptable for sperm viability. Freezing at any time of storage with Bioxell® decreased the sperm viability. However, this effect is significant from 48 h storage onwards (Table I).

The head abnormalities of goat EPS were significantly affected in the model (p=0.013). The effect was appeared after 48 h of cold-storage (Table I, p=0.0092) in both fresh and frozen samples. The sperm tail abnormalities significantly changed in the model (p<0.0001). This abnormality was mainly caused by independent effects of freezing and duration of cold-storage (p<0.0001), i.e., the interaction of both parameters was non-significant (p=0.66). The sperm tail abnormalities were significantly increased from 48 h storage onwards in both fresh and frozen samples (Table I; p<0.0001).

**Figure 1:**
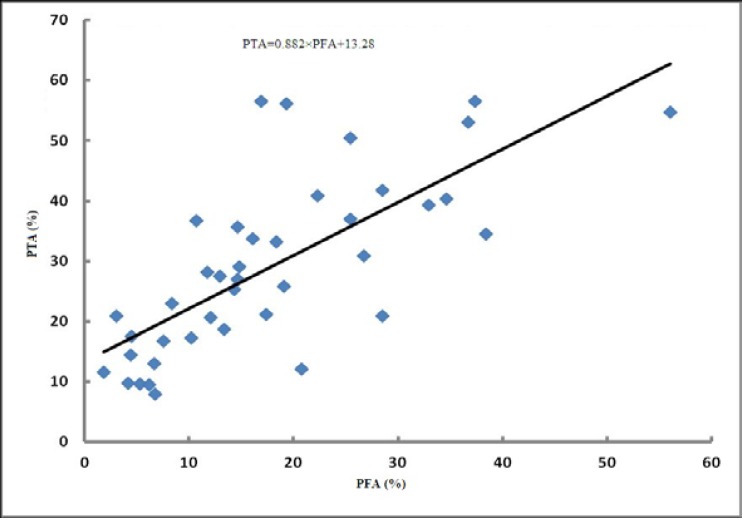
The scatter plot shows a significant linear relationship between the percentages of fresh and frozen sperm abnormalities (p<0.0001). PTA: Post-thaw sperm abnormalities and PFA: pre-freezing sperm abnormalities

The percentage of detached heads was significantly increased by freezing (p=0.0019). Duration of cold-storage (p=0.09) did not influence the abnormality. There was no interaction (p=0.21) between duration of cold-storage and freezing on the percentage of detached heads. While the abnormality in the fresh samples of time 0 h was not affected by freezing, the cold stored sperm lost their heads after freezing, significantly (Table I). A significant regression model was detected between the percentage of head and tail abnormalities before and after freezing as follows (Figure 1);

$\mathrm{PTA}=\left(0.882\times \mathrm{PFA}\right)+13.28$

Where PTA is the percentage of post-thaw sperm abnormalities and PFA is the percentage of pre-freezing sperm abnormalities. 

The percentage of cytoplasmic droplets was significantly affected by freezing, duration of cold-storage and their two-way interaction (p<0.0001). While the percentage of cytoplasmic droplet tend to decrease with increasing time of storage, the respected values significantly declined following cryopreservation at any storage time point (Table I; p<0.0001).

**Table I:** The effects of duration of cold-storage and cryopreservation^†^ on different goat (Caprus hircus) epididymal sperm parameters (L.S.Mean±S.E.M)

		Cold-storage time (h)			
		0	24	48	72
Sperm viability					
	Pre Freezing	94.1 ± 2.53^Aa^	90.8 ± 2.71^Aa^	76.4 ± 2.71^Ab^	79.6 ± 2.75^Ab^
	Post thawing	70.5 ± 2.55^Ba^	65.6 ± 2.56^Ba^	32.7 ± 2.71^Bb^	30.7 ± 2.82^Bb^
Head abnormalities					
	Pre Freezing	0.4 ± 0.27^Aa^	0.57 ± 0.28^Aa^	1.7 ± 0.28^Ba^	0.43 ± 0.29^Aa^
	Post thawing	1.03 ± 0.24^Aa^	0.5 ± 0.29^Aa^	0.9 ± 0.27^Aa^	0.37 ± 0.29^Aa^
Tail abnormalities					
	Pre Freezing	9.04 ± 1.84^Ba^	11.0 ± 1.91^Ba^	17.9 ± 1.95^Bb^	18.9 ± 1.98^Bb^
	Post thawing	17 ± 1.81^Aa^	18.1 ± 1.98^Aa^	28.3 ± 1.91^Ab^	30.2 ± 2.04^Ab^
Cytoplasmic droplets					
	Pre Freezing	46.6 ± 2.51^Ba^	39.8 ± 2.6^Ba^	22.3 ± 2.67^Bb^	20.7 ± 2.7^Bb^
	Post thawing	22.2 ± 2.46^Aa^	15.2 ± 2.7^Ab^	13.8 ± 2.6^Abc^	7.75 ± 2.77^Ac^
Detached heads					
	Pre Freezing	1.7 ± 0.55^Aac^	0.7 ± 0.57^Bab^	2.4 ± 0.58^Ac^	0.94 ± 0.59^Babc^
	Post thawing	1.7 ± 0.54^Aa^	2.4 ± 0.59^Aab^	3.3 ± 0.57^Ab^	3.4 ± 0.61^Ab^
Progressive motility					
	Pre Freezing	86.4 ± 3.63^Aa^	71 ± 3.63^Ab^	51.1 ± 3.63^Ac^	42.2 ± 3.63^Ac^
	Post thawing	27.5 ± 3.63^Ba^	28.5 ± 3.63^Ba^	16.7 ± 3.63^Bb^	10.2 ± 3.63^Bb^

^abc^ Values with different superscripts within rows significantly differ (p<0.05).

^ABC^ Values with different superscript within column significantly differ (p<0.05).

^†^Ten pairs of testes-epididymes within each storage time group. Two straws per storage time were used for frozen sperm analysis.

## Discussion

The results of the present study showed that long time (48 h onwards) in situ cold-storage reduces viability and progressive motility and increases abnormalities of goat EPS. Different studies highlighted the impact of postmortem time to sperm recovery on the EPS parameters (1, 19). Elongation of postmortem time to sperm recovery alters the chemical composition and reduces pH of epididymal lumen, and in turn reduces the sperm quality (9). Cold-storage postpones the beginning of the postmortem changes and may protect EPS (1, 9, 20). 

However, the EPS response to cold-storage depends on species (7, 20). Elongated post-mortal time to sperm recovery in Spanish ibex did not influence DNA integrity of EPS (1). In the present study, freezing significantly reduced the sperm viability, progressive motility and increased sperm abnormalities. The responses of cold-stored EPS to freezing were similar at all storage-time points, except detached heads. The caprine EPS was successfully cryopreserved and some attempts to develop a suitable extender for the goat EPS cryopreservation were reported (-). 

Blash *et al* reported the first successful freezing of the goat EPS compared to the ejaculated semen (21). To optimize a freezing medium, Kundu *et al* found that modified ringer solution with DMSO and ethylene glycol may not protect the goat EPS against freezing damage (23). Addition of dextran and amino acids to modified ringer solution improved sperm progressive motility of goat EPS following freezing (,  24). 

Some studies on freezing of the wild species of goat (Spanish ibex) EPS have been documented. Santiago-Moreno et al found that chicken egg yolk in tris-citric acid- glucose-lactose solution can improve freezing of goat EPS specially with 6% (v/v) (25, 26). The present study addressed the effect of an environmental insult, i.e. freezing of cold-stored sperm, on the percentage detached heads. It means while the minimum changes within epididymal lumen occur, the retrieved sperm will be sensitive to freezing in term of increase detached heads. There are different reports on the possible pathogenesis of this defect. 

The long time exposure to chemicals or additives disrupts the spermatogenesis and causes the defect (-). A genetic based etiology has been considered as a cause of the defect, too (-). However, the reports of increased rate of detached heads in senesce sperm and in vesiculectomized bulls confirm the effect of the environment as an important predisposing factor (33-35). Freezing as well as the duration of cold-storage (up to 72 h) reduced the percentage of cytoplasmic droplets. The possible mechanism for cytoplasmic droplet loss in ejaculated sperm is exposure to seminal vesicle fluid, which has a different electrolyte composition to that of epididymal lumen (36). 

During cryopreservation, sperm has to tolerate hyperosmotic solutions and pass the crystal formation temperature zone: Which factor cause cytoplasmic droplet to loss, is not known. Alteration in the contents of epididymal lumen during cold-storage may contribute in droplet loss in the present study. 

## Conclusion

In conclusion, the goat EPS was stored in refrigerator (4^o^C) for 72h with acceptable progressive motility and viability. However, cold-storage may not protect the goat EPS against cryopreservation damages with Bioxell.
